# Black Soldier Fly-Composted Organic Fertilizer Enhances Growth, Yield, and Nutrient Quality of Three Key Vegetable Crops in Sub-Saharan Africa

**DOI:** 10.3389/fpls.2021.680312

**Published:** 2021-06-02

**Authors:** Abel O. Anyega, Nicholas K. Korir, Dennis Beesigamukama, Ghemoh J. Changeh, Kiatoko Nkoba, Sevgan Subramanian, Joop J. A. van Loon, Marcel Dicke, Chrysantus M. Tanga

**Affiliations:** ^1^International Centre for Insect Physiology and Ecology, Nairobi, Kenya; ^2^Department of Agricultural Science and Technology, Kenyatta University, Nairobi, Kenya; ^3^Department of Crop Production and Management, Busitema University, Tororo, Uganda; ^4^Centre for African Bio-Entrepreneurship (CABE), Nairobi, Kenya; ^5^Laboratory of Entomology, Plant Sciences Group, Wageningen University, Wageningen, Netherlands

**Keywords:** black soldier fly frass fertilizer, nitrogen uptake, nitrogen use efficiency, nutritional quality, soil health, vegetable productivity

## Abstract

Worldwide, French beans (*Phaseolus vulgaris* L.), tomato (*Solanum lycopersicum* L.), and kales (*Brassica oleracea* L. *var. acephala*) are considered economically important food crops. There is a rapid decline in their yield due to severe soil degradation. Thus, high commercial fertilizer inputs are crucial, though they remain expensive and inaccessible to resource poor farmers. We investigated the comparative performance of composted black soldier fly frass fertilizer (BSFFF), conventionally composted brewer's spent grain (BSG), commercial organic fertilizer (Evergrow), and mineral [nitrogen, phosphorus, and potassium (NPK)] fertilizer on growth, yield, N use efficiency, and nutritional quality (crude protein, crude fiber, crude fats, ash, and carbohydrate concentrations) of tomatoes, kales, and French beans under greenhouse and open-field conditions for two seasons. The fertilizers were applied at rates equivalent to 371 kg of N ha^−1^. For each crop, the plots were treated with sole rates of BSFFF, BSG, Evergrow, and NPK to supply 100% of the N required. Additional treatments included a combination of BSFFF and NPK, and BSG and NPK so that each fertilizer supplies 50% of the N required. The control treatment consisted of unfertilized soil. Results show that vegetable yields achieved using a combination of BSFFF and NPK were 4.5, 2.4, and 5.4-folds higher than the yield from the control treatment for tomatoes, kales, and French beans, respectively. The combined application of BSFFF and NPK produced 22–135%, 20–27%, and 38–50% higher yields than sole NPK for tomatoes, kales, and French beans, respectively, under both greenhouse and open-field conditions. The highest agronomic N use efficiency was achieved in sole BSFFF-treated plots compared to sole BSG and Evergrow. The N taken up by the vegetables was significantly higher when BSFFF and NPK were integrated. Vegetables grown using a combination of BSFFF and NPK had the highest crude protein and ash concentrations. Our findings demonstrate that the integration of BSFFF and NPK in vegetable cropping systems at the recommended rate of 1.24 t ha^−1^ BSFFF and 322 kg ha^−1^ NPK would improve soil health, boost yield, and nutritional quality of vegetable crops.

## Introduction

Horticulture is an important sector because it contributes to food and nutrition security, household income, employment, and foreign exchange in Sub-Saharan Africa (SSA) (Frank et al., 2019; Amao, 2020; Ebert, 2020; Minten et al., 2020). Vegetables, such as tomatoes, kales, and French beans, are high-value crops grown in most African countries including Kenya (van der Lans et al., 2012; Cernansk, 2015; Nordey et al., 2017). Tomatoes (*Solanum lycopersicum* L.) are highly nutritious and rich in vitamins A, B, and C and also lycopene, an antioxidant linked to a reduction of diseases like cancer (Dorais et al., 2008; Capanoglu et al., 2010). Kales (*Brassica oleracea* L.) contain high levels of carotenoids, minerals, and prebiotic carbohydrates, making them a healthy food source (Migliozzi et al., 2015). French beans (*Phaseolus vulgaris* L.) also known as green beans or snap beans have a significant amount of minerals, vitamins, and proteins (Messina, 2014; Celmeli et al., 2018), which are vital for human nutrition. Therefore, vegetable consumption is crucial in offering protection against non-communicable diseases, thus improving human health.

Despite their nutritional and economic values, the vegetable production in SSA is highly affected by low soil fertility (Tully et al., 2015; Mantovani et al., 2017). Most soils in SSA are deficient in macronutrients and secondary nutrients [calcium (Ca), magnesium (Mg), and sulfur (S)] (Gachimbi et al., 2005; Nkonya et al., 2008; Cobo et al., 2010; Wortmann et al., 2019) due to soil erosion and leaching losses (Alfaro et al., 2008) and yet, very little (≤ 10 kg ha^−1^ per year) or no mineral fertilizer is used (FAO, 2017). Most soils in Kenya are low in organic matter (<3%) (Gachimbi et al., 2005) and exhibit high acidity (Keino et al., 2015).

Organic fertilizers improve soil microbial activities and provide macronutrients necessary for the growth of crops (Alfaro et al., 2008; Islam et al., 2017). In addition, organic fertilizers supply secondary nutrients (Ca, Mg, and S) and micronutrients (zinc, boron, and manganese), which play a key role in the uptake and utilization of macronutrients [mostly nitrogen (N), phosphorous (P), and potassium (K)] (Tittonell et al., 2008a; Wortmann et al., 2019). A combined application of organic and inorganic fertilizers has been recommended to improve and sustain soil fertility, crop yields, and agronomic nutrient use efficiency in SSA (Tittonell et al., 2008b; Vanlauwe et al., 2014, 2015). Despite their critical role in the restoration of natural fertility, most farmers in SSA do not apply organic fertilizers because most organic resources have other competing uses such as fuel and feeding livestock on the farm (Rufino et al., 2011; Ndambi et al., 2019).

Insect mass rearing systems produce huge quantities of frass (a combination uneaten substrate, feces, and exuviae) with a great potential for improving soil and crop productivity (Kagata and Ohgushi, 2012; Poveda et al., 2019; Beesigamukama et al., 2020b; Houben et al., 2020; Quilliam et al., 2020; Menino et al., 2021). Black soldier fly (BSF) (*Hermetia illucens* L.) frass fertilizer (BSFFF) is a byproduct from bioconversion of organic wastes using BSF larvae into nutrient-rich and hygienic organic fertilizer (Erickson et al., 2004; Lalander et al., 2015, 2016). Unlike the conventional composting process, which takes 8–24 weeks, BSF-assisted composting takes only 5 weeks to convert organic waste into mature and stable organic fertilizer (Beesigamukama et al., 2021). The high bioconversion efficiency of BSF could be partly attributed to the high waste reduction efficiency (65–79%) (Diener et al., 2011) and the high abundance and diversity of microbial decomposers associated with BSF larvae (Elhag et al., 2017; Vogel et al., 2018). Recent studies have reported improved crop growth, yield, nutrient uptake, N use efficiency, and disease suppression in different plants grown using composted BSFFF (Choi and Hassanzadeh, 2019; Beesigamukama et al., 2020b,c; Quilliam et al., 2020).

Most research on the use of BSFFF has been conducted using non-vegetable crops (Beesigamukama et al., 2020b,c; Menino et al., 2021), except Quilliam et al. (2020) who used chili pepper and shallots, but this study did not investigate the nutritional quality of the crops involved. It should be noted that different crops have different nutrient requirements, and thus require different fertilizer application rates. The BSFFF is a relatively new product, and information on its performance in terms of how it influences the growth, yield, and nutritional quality of widely consumed vegetables, such as tomatoes, kales, and French beans, is largely unknown.

The performance of BSFFF for tomatoes, kales, and French beans production in comparison to existing organic fertilizers has not been studied. The combined application of organic and mineral fertilizers has been recommended for improved nutrient use efficiency and crop yield (Vanlauwe et al., 2015). However, information on the agronomic performance and nutritional quality of vegetables grown with sole BSFFF and a combination of BSFFF and mineral fertilizers is lacking. Therefore, the current study aimed to determine the effects of BSFFF on the growth, yield, N uptake and use efficiency, and nutritional quality of tomatoes, kales, and French beans in comparison with conventional compost, and commercial organic and mineral fertilizers.

## Materials and Methods

### Experimental Site

Greenhouse and field experiments were carried out in Nairobi County, Kenya. The greenhouse experiment was conducted at the International Centre of Insect Physiology and Ecology (icipe) (36.89°E, 1.17°S), whereas the field experiment was carried out at Kenyatta University teaching and demonstration farm (36.94°E, 1.18°S) for two cropping seasons (October to March 2018 and May to September 2019). Nairobi receives bimodal rainfall with an annual average amount of 787 mm. The long rain season falls between March and May, whereas the short rain season runs from October to December. The temperature of Nairobi ranges between 12 and 29°C. The soils in the study sites are Acric Ferralsols (Gachene and Kimaru, 2003) with medium acidity (5.6–5.8), low levels of N (0.05–0.13%), phosphorus (5–9 ppm), and organic matter (0.4–2.1%) (Table 1). Before experiments, soil samples were collected from 0 to 20 cm depth, air-dried for 5 days, and analyzed for nutrients, pH, organic matter, and texture using standard laboratory methods described in Okalebo et al. (2002). Table 1 presents the results of the soil analysis.

**Table 1:** Selected characteristics of soils used during green house and open-field experiments.

**Parameter**	**Greenhouse**	**Open field**
pH	5.80	5.59
Total nitrogen (%)	0.05	0.13
Total organic carbon (%)	0.24	1.23
Organic matter (%)	0.42	2.1
Available phosphorus (ppm)	5.0	9.0
Potassium (cmol kg^−1^)	0.84	1.44
Calcium (cmol kg^−1^)	2.2	6.0
Magnesium (cmol kg^−1^)	2.77	3.85
Manganese (cmol kg^−1^)	0.43	0.48
Copper (ppm)	1.00	1.45
Iron (ppm)	29.5	44.4
Zinc (ppm)	3.00	2.47
Sodium (cmol kg^−1^)	0.28	1.19
Textural class	Loam	Sandy loam

### Source of Fertilizers

The experiment involved four fertilizers: composted BSFFF, conventionally composted organic fertilizer [brewer's spent grains (BSGs)], commercial organic fertilizer (Evergrow), and mineral fertilizer (NPK 23:23:0). The Evergrow and NPK were sourced from Sanergy and Kenya Farmers Association Ltd., respectively, all located in Nairobi, Kenya. The BSFFF was obtained frass generated from the feeding of BSF larvae on BSG at the animal rearing and quarantine unit at icipe. The BSF larvae were reared following procedures described by Beesigamukama et al. (2021). The BSG compost was generated from the composting process of BSG sourced from Kenya Breweries Limited, Nairobi, Kenya. The BSF frass and BSG were composted separately for 112 days using the heap composting method. Before the start of composting, the C/N ratios of BSF frass and BSGs were adjusted to 25:1 (Strong, 1911) using rice husks. The amounts of rice husk required to adjust C/N ratios of BSF frass and BSGs were calculated using a formula given by Richard and Trautmann (1996). The frass obtained from BSF rearing and BSG were composted separately to convert into mature and stable compost products.

During composting, heaps of 1 m height and 4 m long were built on surfaces lined with polythene sheets and hydrated to a moisture content of 55–65% (Chen et al., 2009). The heaps were covered using polythene sheets (1,000 mm gauge) to prevent moisture and heat losses. The composting materials were turned on a weekly basis using a forked spade to ensure uniform decomposition. Compost maturity was monitored biweekly up to maturity stage using temperature, pH (6–8), C/N ratio (<25), germination index (>80%) (Bernal et al., 2009). A phytotoxicity test was conducted using cabbage seeds (*Brassica oleracea var. capitata*) following protocols described by Emino and Warman (2004). Table 2 presents selected physical–chemical characteristics (dry weight basis) of mature composts and the commercial organic fertilizer used in experiments.

**Table 2:** Characteristics of organic fertilizers used during experiments.

**Parameter**	**Composted BSF frass fertilizer (BSFFF)**	**BSG compost**	**Evergrow fertilizer**
pH	7.26	7.11	7.8
Organic carbon (%)	38.61	37.9	20
Nitrogen (%)	3.61	2.45	1.0
Phosphorus (%)	0.50	0.37	0.4
Potassium (%)	0.29	0.24	0.5
Calcium (%)	0.97	0.28	1.3
Magnesium (%)	0.10	0.15	0.2
Iron (ppm)	310	185	0.12
Copper (ppm)	25.0	15.0	100
Manganese (ppm)	109	262	200
Zinc (ppm)	182	167	850
C/N ratio	10.7	15.5	20
Germination index (%)	86	75	–

### Treatments and Experimental Setup

The BSFFF, BSG, Evergrow, and NPK fertilizer were applied at uniform rates equivalent to 371 kg N ha^−1^ in two sets of treatments. In the first set of treatments, 100% of the N required was supplied using sole organic fertilizers and sole NPK; these were denoted as BSFFF, BSG, and Evergrow, NPK, respectively. The quantity of fertilizers required to supply the 371 kg N ha^−1^ were equivalent to 2.5, 3.5, and 14.8 t ha^−1^ for sole BSFFF, BSG, and Evergrow, respectively, and 644.7 kg ha^−1^ for sole NPK. In the second set of treatments, the BSFFF and BSG were combined with mineral fertilizer, whereby 50% of the N required was supplied using organic fertilizers, and the other 50% was supplied using mineral NPK fertilizer. These were denoted as BSFFF + NPK and BSG + NPK, respectively. The quantities of fertilizers required to supply 50% of the N required (that is 185.5 kg N ha^−1^) were equivalent to 1.24, 1.73 t ha^−1^ for BSFFF and BSG, respectively, and 322.3 kg ha^−1^ for NPK. The control treatment consisted of unfertilized soil.

The experiments were carried out under greenhouse and open-field conditions. The greenhouse experiment was performed using polythene pots measuring 30 cm long, 40 cm wide, and 60 cm deep. Each polythene pot was filled with 10 kg of fresh soil, which was mixed with the different treatments. The experiments were arranged in a randomized complete block design (RCBD) with four replicates. Each replicate consisted of 10 pots, arranged at a spacing of 60 cm × 60 cm, 45 cm × 45 cm, and 30 cm × 30 cm for tomatoes, kales, and French beans, respectively.

In the field experiment, two 14-day-old seedlings of tomato (variety: Kilele) and kale (variety: Southern Georgia) were planted per hill, whereas two French beans seeds (variety: Samantha) were sown per hill. At 2 weeks after germination, thinning was done to maintain one plant per pot and hill for the greenhouse and field experiments, respectively. The field experiment was carried out using plots measuring 2.5 m × 2.5 m with borders of 0.5 m. The organic fertilizers (BSFFF, BSG, and Evergrow) were applied 1 week before sowing, whereas the mineral fertilizer (NPK) was applied 1 week after planting. During experiments, weeds were controlled manually by pulling, whereas a drip irrigation system was installed to cater for crop water requirements.

### Vegetable Growth, Yield, and Nutritional Quality

Six vegetable plants were randomly selected and tagged from each treatment replicate for the determination of growth parameters. Plant growth for tomatoes and kales was determined by collecting data on plant height, stem diameter, and number of leaves biweekly from the 4th up to 10th week of experiments. For French beans, growth data were collected weekly from the 4th to 7th week of experiments. Plant height was measured using a tape measure placed from ground level to the tip of the shoot. The stem diameter was measured using a Vernier caliper placed at the 5 cm mark from the ground level. Tomato yield was determined by picking mature fruits, whereas kale yield was determined by picking mature leaves from the 7th week until the end of experiments. For French bean, pods were collected on a weekly basis from the 5th week until the end of experiments. The total vegetable yield per crop was determined by summing the yields obtained per harvest period and expressed on hectare basis (t ha^−1^).

Vegetable yield from the field experiments was used to calculate the N uptake (Equation 1) and agronomic N efficiency (AE_N_), which is a measure of economic yield produced per unit amount of N supplied from each treatment (Equation 2) (Baligar et al., 2001).

(1) $$\begin{array}{l} \text{Nitrogen uptake }\left(\text{kg N h}{\text{a}}^{-1}\right)\\ =\frac{\left[\text{N}\left(\text{\%}\right)\times \text{dry matter}\left(\text{kg h}{\text{a}}^{-1}\right)\right]}{100} \end{array}$$

(2) $$\begin{array}{l} \text{A}{\text{E}}_{\text{N}}\left(\text{kg k}{\text{g}}^{-1}\text{N}\right)\\ =\frac{\left[\text{Crop yiel}{\text{d}}_{\text{F}}\left(\text{kg h}{\text{a}}^{-1}\right)-\text{Crop yiel}{\text{d}}_{\text{C}}\left(\text{kg h}{\text{a}}^{-1}\right)\right]}{\text{Quantity of N applied }\left(\text{kg h}{\text{a}}^{-1}\right)} \end{array}$$

where *F* represents fertilizer-treated plot and *C* represents the control plot (no amendment).

A part of the tomato fruits, kale leaves, and French bean pods harvested from the field experiment were air-dried and ground into powder using an analytical mill to analyze for total N crude protein (total N using the Kjeldahl method followed which was converted to crude protein using a factor of 6.25), crude fiber (Velp fiber analyzer, VELP Scientifica, Usmate Velate, Italy), crude fat (Velp fat analyzer, VELP Scientifica, Usmate Velate, Italy) (Van Soest et al., 1991), and carbohydrates (Association of Official Analytical Chemists, 1990), whereas the ash concentration was calculated (Association of Official Analytical Chemists, 1990).

### Data Analysis

Prior to analysis, data were checked for normality using the Shapiro–Wilk test. Plant height, number of leaves, and stem diameter were analyzed using a linear mixed-effect model with “lmer” function from the package “lme4” in R programming environment (R Core Team, 2019) with fertilizer treatment and sampling time as fixed effects, and replication as random effect. Data on crop yields, N accumulation AE_N_, and nutritional quality were analyzed using the ANOVA test. Mean separation was done using the Student–Newman–Keuls test with α = 0.05. The principal component analysis (PCA) was performed using the “prcomp” (Venables and Ripley, 2002) function from the “ggbiplot” (Wickham, 2016) package to examine the relationship among the growth, yield, and nutritional quality parameters of vegetables grown using different fertilizer treatments. Data were analyzed separately for each crop. All the statistical analyses were conducted using the R programming environment (R Core Team, 2019).

## Results

### Vegetable Growth

#### Tomato

The tomato plant height varied significantly due to fertilizer treatments (greenhouse: *p* < 0.001, open field: *p* < 0.001). The plant height under greenhouse and open-field conditions followed an increasing trend throughout experiments (Table 3). Application of BSFFF + NPK produced significantly (*p* < 0.001) taller tomato plants than where BSG was applied. Amendment with BSFFF + NPK produced tomato plants, which were significantly (*p* < 0.001) taller than those grown using BSG, NPK, and Evergrow fertilizers. All fertilizer-treated plots produced significantly (*p* < 0.001) taller tomato plants than the control treatment.

**Table 3:** Effect of composted BSF frass, conventionally composted BSG, and commercial fertilizers on the growth of tomatoes.

**Parameter**	**Treatments**	**Greenhouse**				**Open field**			
		**4 weeks**	**6 weeks**	**8 weeks**	**10 weeks**	**4 weeks**	**6 weeks**	**8 weeks**	**10 weeks**
Plant height (cm)	BSFFF	30.8 ± 2.89ab	53.8 ± 3.86b	71.0 ± 3.02ab	99 ± 4.75ab	34.8 ± 1.49ab	52.6 ± 2.46bc	74.0 ± 3.86b	84.2 ± 4.22ab
	BSG	24.3 ± 1.20c	39.0 ± 1.59c	63.0 ± 4.62b	76.3 ± 6.59b	28.7 ± 1.79cd	44.0 ± 2.22d	57.1 ± 2.29c	70.8 ± 3.07c
	NPK	33.6 ± 1.15ab	60.0 ± 4.59ab	73.0 ± 5.78ab	91.0 ± 3.44bc	39.7 ± 1.85a	56.1 ± 1.6ab	65.7 ± 3.21bc	75.8 ± 2.9bc
	Evergrow	32.1 ± 1.23ab	50.0 ± 2.60b	65.8 ± 3.42b	74.0 ± 4.61c	32.4 ± 1.88bc	46.4 ± 3.24cd	58.3 ± 3.72c	66.8 ± 3.19c
	BSFFF + NPK	35.7 ± 1.17a	68.2 ± 3.56a	86.0 ± 4.68a	112.9 ± 3.8a	38.4 ± 1.69ab	62.1 ± 2.18a	85.8 ± 3.80a	90.2 ± 2.59a
	BSG + NPK	29.1 ± 1.20b	51.6 ± 2.73b	78.0 ± 4.62ab	100.4 ± 7.0ab	35.7 ± 1.43ab	54.5 ± 2.76abc	69.5 ± 2.76b	86.4 ± 2.5ab
	Control	18.8 ± 1.20d	26.6 ± 3.92d	37.0 ± 4.12c	49.1 ± 4.55d	24.8 ± 1.73d	35.2 ± 2.40e	40.3 ± 2.56d	47.1 ± 3.05d
Number of leaves	BSFFF	9.0 ± 0.54a	12 ± 1.11a	14.6 ± 0.87a	16.5 ± 1.17a	8.3 ± 0.37a	10.0 ± 0.27b	12.3 ± 0.67abc	13.5 ± 0.78abc
	BSG	7.6 ± 1.01a	10 ± 1.15a	10.8 ± 1.11b	12.3 ± 0.91b	6.3 ± 0.25b	8.8 ± 0.25c	10.5 ± 0.53c	11.5 ± 0.42c
	NPK	7.8 ± 0.59a	10.9 ± 0.9a	13.4 ± 0.98ab	15.3 ± 0.97ab	9.0 ± 0.27a	10.9 ± 0.4ab	11.8 ± 0.56bc	12.8 ± 0.56bc
	Evergrow	8.5 ± 0.87a	9.6 ± 0.85a	10.5 ± 1.19b	12.8 ± 1.21b	8.8 ± 0.25a	10.5 ± 0.19b	11.5 ± 0.46bc	12.0 ± 0.38c
	BSFFF + NPK	8.6 ± 0.66a	11.6 ± 0.69a	15.4 ± 0.69a	17.5 ± 0.59a	9.0 ± 0.27a	12.1 ± 0.52a	13.9 ± 0.58a	15.3 ± 0.59a
	BSG + NPK	8.9 ± 1.16a	10.6 ± 1.1a	13.8 ± 0.82ab	16.2 ± 1.15a	8.5 ± 0.33a	11.4 ± 0.53ab	13.3 ± 0.7ab	14.5 ± 0.71ab
	Control	5.6 ± 0.39a	6.6 ± 0.18b	7.1 ± 0.27c	8.1 ± 0.23c	5.6 ± 0.26b	7.0 ± 0.27d	8.1 ± 0.30d	9.1 ± 0.35d
Stem diameter (mm)	BSFFF	7.9 ± 0.88a	10.2 ± 0.7a	12.9 ± 1.15a	14.0 ± 1.16ab	6.3 ± 0.81ab	9.1 ± 0.74a	11.1 ± 0.79ab	12.4 ± 0.75ab
	BSG	7.9 ± 0.46a	9.4 ± 0.45a	10.2 ± 0.65a	11.0 ± 0.78b	5.6 ± 0.54ab	8.1 ± 0.66a	8.9 ± 0.72b	9.8 ± 0.73c
	NPK	8.8 ± 0.24a	10.4 ± 0.22a	11.9 ± 0.70a	12.9 ± 0.8ab	7.4 ± 0.54a	8.9 ± 0.65a	10.1 ± 0.71ab	11.4 ± 0.76bc
	Evergrow	8.4 ± 0.56a	9.5 ± 0.43a	10.5 ± 0.62a	11.5 ± 0.78b	6.4 ± 0.64ab	7.6 ± 0.73a	8.5 ± 0.78b	9.6 ± 0.89c
	BSFFF + NPK	8.3 ± 0.87a	10.9 ± 1.02a	14.0 ± 1.28a	15.6 ± 1.06a	6.9 ± 0.73a	10.0 ± 0.79a	12.6 ± 0.6a	14.2 ± 0.7a
	BSG + NPK	7.3 ± 0.68a	10.8 ± 0.89a	12.4 ± 0.96a	13.6 ± 0.93ab	6.9 ± 0.77a	9.9 ± 0.75a	11.2 ± 0.66ab	13.0 ± 0.7ab
	Control	4.6 ± 0.67b	5.2 ± 0.76b	6.0 ± 0.70b	6.8 ± 0.62c	4.0 ± 0.64b	4.7 ± 0.67b	5.6 ± 0.68c	6.2 ± 0.67d

*BSFFF, black soldier fly frass organic fertilizer applied at sole rate equivalent to 371 kg N ha^−1^; BSG, conventional compost from brewer's spent grain applied at sole rate equivalent to 371 kg N ha^−1^; NPK, commercial mineral fertilizer applied at sole rate equivalent to 371 kg N ha^−1^; Evergrow, commercial organic fertilizer applied at sole rate equivalent to 371 kg N ha^−1^; BSFFF + NPK, combined application of BSFFF and NPK so that each supplies 185.5 kg N ha^−1^; BSG + NPK, combined BSG compost and NPK so that each supplies 185.5 kg N ha^−1^, control, unfertilized soil. Within the same column and per parameter, means (±SE) followed by the same letters are not significantly different for α = 0.05*.

The number of leaves varied significantly (*p* < 0.001) due to different fertilizer treatments. The number of leaves increased throughout experiments (Table 3). Tomatoes grown using fertilizer amendments produced a significantly (*p* < 0.001) higher number of leaves than those grown using the unfertilized soil. The soil treated with BSFFF produced a significantly (*p* < 0.001) higher number of tomato leaves than those grown using BSG and Evergrow at 8 and 10 weeks after planting under greenhouse conditions. Amendment with BSFFF + NPK produced significantly (*p* < 0.001) higher number of leaves than NPK, Evergrow, and BSG from the 8th to 10th week after planting under open-field conditions.

The tomato stem diameter also varied significantly due to different fertilizer treatments during greenhouse (*p* < 0.001) and open-field (*p* < 0.001) experiments. The stem diameter followed an increasing trend throughout experiments. Fertilizer treatments produced plants with significantly (*p* < 0.001) higher values compared to the control treatment (Table 3). During open-field experiments, tomatoes grown using BSFFF + NPK had significantly (*p* < 0.001) bigger stem diameter than those produced using Evergrow and BSG at 8 weeks after planting, and where BSG, NPK, and Evergrow were applied at 10 weeks after planting.

#### Kales

The height of kales varied significantly due to different fertilizer treatments (greenhouse: *p* < 0.001, open field: *p* < 0.001) (Table 4). The plant height increased to peak values at 10 weeks after planting. It was noted that fertilizer-treated soils produced significantly (*p* < 0.001) taller plants than the control. At 6 weeks after planting, amendment with NPK produced significantly (*p* < 0.001) taller plants than where Evergrow was applied under greenhouse experiments. Under open-field conditions, plots treated with BSFFF + NPK produced significantly (*p* < 0.001) higher plant than other treatments, except where BSG + NPK, NPK, and BSFFF were applied at 4 weeks after planting, and BSG + NPK and BSFFF at 8 weeks after planting.

**Table 4:** Effect of composted BSF frass, conventionally composted BSG, and commercial fertilizers on the growth of kales.

**Parameter**	**Fertilizer treatment**	**Greenhouse**				**Open field**			
		**4 weeks**	**6 weeks**	**8 weeks**	**10 weeks**	**4 weeks**	**6 weeks**	**8 weeks**	**10 weeks**
Plant height (cm)	BSFFF	14.7 ± 1.96a	21.3 ± 1.81ab	34.1 ± 1.56a	36.3 ± 2.37a	12.7 ± 0.96ab	20.9 ± 1.17b	28.0 ± 1.44ab	30.4 ± 1.24b
	BSG	14.8 ± 1.25a	20.1 ± 2.5ab	29.6 ± 0.56a	33.3 ± 1.3a	11.0 ± 0.63b	16.7 ± 0.71c	23.2 ± 0.93c	26.1 ± 1.07cd
	NPK	15.4 ± 2.42a	27.7 ± 0.73a	30.0 ± 0.5a	33.6 ± 0.49a	12.5 ± 0.6ab	19.8 ± 0.81b	25.7 ± 1.32bc	28.5 ± 1.28bc
	Evergrow	15.6 ± 1.55a	19.0 ± 1.82b	27.5 ± 1.99a	31.1 ± 1.2a	11.2 ± 0.73b	18.2 ± 1.01bc	22.3 ± 1.12c	24.1 ± 1.34d
	BSFFF + NPK	16.4 ± 1.41a	25.5 ± 2.7ab	35.2 ± 1.91a	38.5 ± 1.52a	14.8 ± 0.52a	24.4 ± 0.83a	29.9 ± 0.93a	33.8 ± 0.94a
	BSG + NPK	14.5 ± 1.28a	23.9 ± 2.24ab	32.6 ± 2.98a	35.9 ± 1.93a	13.5 ± 0.56ab	21.1 ± 0.81b	27.7 ± 0.9ab	30.1 ± 0.88b
	Control	9.6 ± 0.56b	11.4 ± 0.77c	17.8 ± 2.37b	18.2 ± 2.34b	8.0 ± 0.34c	11.9 ± 0.79d	15.9 ± 0.9d	18.8 ± 0.88e
Number of leaves	BSFFF	6.9 ± 0.8a	12.2 ± 1.24a	15.2 ± 1.03a	15.9 ± 0.96ab	9.0 ± 0.7a	11.7 ± 0.74ab	14.3 ± 0.9ab	15.4 ± 0.8ab
	BSG	5.4 ± 0.86a	11.3 ± 1.48ab	13.2 ± 1.07ab	14.0 ± 1.09ab	7.5 ± 0.7a	10.5 ± 0.75b	12.5 ± 0.72b	13.9 ± 0.78b
	NPK	6.5 ± 1.1a	9.2 ± 1.14ab	13.3 ± 0.79ab	14.1 ± 0.74ab	10.0 ± 0.74a	12.0 ± 0.73ab	14.0 ± 0.84ab	14.9 ± 0.85ab
	Evergrow	8.2 ± 1.11a	10.5 ± 0.72ab	11.6 ± 0.42b	12.6 ± 0.5b	9.0 ± 0.69a	11.5 ± 0.75ab	12.6 ± 0.67b	13.2 ± 0.71ab
	BSFFF + NPK	7.3 ± 1.14a	12.2 ± 1.11a	15.8 ± 1.13a	17.1 ± 1.19a	9.6 ± 0.6a	13.9 ± 0.68a	16.2 ± 0.74a	16.8 ± 0.77a
	BSG + NPK	8.2 ± 1.17a	10.5 ± 1.13ab	15.0 ± 0.68ab	16.2 ± 0.64ab	9.6 ± 0.67a	12.3 ± 0.93ab	15.2 ± 0.87ab	15.8 ± 0.86ab
	Control	5.1 ± 0.76a	7.1 ± 0.78ab	8.8 ± 0.73c	9.7 ± 0.84c	5.5 ± 0.67b	7.1 ± 0.69c	8.9 ± 0.73c	9.8 ± 0.72c
Stem diameter (mm)	BSFFF	7.4 ± 1.01a	13.3 ± 1.09ab	18.1 ± 1.78ab	20.8 ± 2.03ab	9.5 ± 0.71bc	14.8 ± 0.75bc	18.8 ± 1.23ab	21.2 ± 1.27a
	BSG	6.3 ± 1.44a	10.9 ± 1.8ab	14.9 ± 0.81b	16.8 ± 1.0bc	8.9 ± 0.75c	12.5 ± 0.67c	15.6 ± 0.71c	16.5 ± 0.69b
	NPK	8.5 ± 1.09a	10.7 ± 0.7ab	14.7 ± 1.16b	17.7 ± 1.54bc	12.3 ± 0.73ab	14.9 ± 0.7bc	17.0 ± 0.73bc	18.2 ± 0.74b
	Evergrow	8.1 ± 1.6a	9.5 ± 1.16ab	13.5 ± 1.11b	15.1 ± 1.09c	11.0 ± 0.75abc	13.4 ± 0.6bc	14.9 ± 0.58c	15.4 ± 0.58b
	BSFFF + NPK	9.1 ± 2.05a	14.8 ± 2.05a	19.9 ± 0.52a	23.1 ± 1.14a	12.6 ± 0.72a	19.0 ± 1.25a	21.4 ± 1.15a	22.3 ± 1.12a
	BSG + NPK	8.2 ± 1.01a	11.8 ± 1.69ab	15.8 ± 1.11b	18.8 ± 1.2abc	11.3 ± 0.85abc	15.8 ± 0.56b	19.2 ± 0.36ab	21.0 ± 0.69a
	Control	5.3 ± 0.87a	7.3 ± 1.21b	7.3 ± 1.06c	10.1 ± 1.07d	6.4 ± 0.73d	8.0 ± 0.7d	9.3 ± 0.68d	10.3 ± 0.77c

*BSFFF, black soldier fly frass organic fertilizer applied at sole rate equivalent to 371 kg N ha^−1^; BSG, conventional compost from brewer's spent grain applied at sole rate equivalent to 371 kg N ha^−1^; NPK, commercial mineral fertilizer applied at sole rate equivalent to 371 kg N ha^−1^; Evergrow, commercial organic fertilizer applied at sole rate equivalent to 371 kg N ha^−1^; BSFFF + NPK, combined application of BSFFF and NPK so that each supplies 185.5 kg N ha^−1^; BSG + NPK, combined BSG compost and NPK so that each supplies 185.5 kg N ha^−1^, control, unfertilized soil. Within the same column and per parameter, means (±SE) followed by the same letters are not significantly different for α = 0.05*.

The number of kale leaves was observed to vary significantly (greenhouse: *p* < 0.001, open field: *p* < 0.001) due to fertilizer amendments (Table 4). The number of kale leaves reached peak values at 10 weeks after planting, with minimal changes observed between the 8th and 10th week. Fertilizer-treated plots produced kale plants with a significantly (*p* < 0.001) higher number of leaves than the control treatment, except at 4 and 6 weeks during greenhouse experiments. At 8 and 10 weeks after planting, amendment with BSFFF + NPK produced significantly (*p* < 0.001) higher number of kale leaves than where Evergrow was applied during greenhouse experiments. During the open-field experiments, kales grown in plots treated with both BSFFF + NPK had a significantly (*p* < 0.001) higher number of leaves than those grown using BSG from the 8th to 10th week after planting, and Evergrow at 8 weeks after planting.

The stem diameter of kales grown using different fertilizer treatments varied significantly (greenhouse: *p* < 0.001, open field: *p* < 0.001) during experiments. Peak values of stem diameter were observed 10 weeks after planting (Table 4). Amendment with BSFFF + NPK fertilizers produced the biggest stem, which was significantly (*p* < 0.001) bigger than those of other treatments, except BSFFF at 8 and 10 weeks after planting and BSG + NPK at 10 weeks after planting under greenhouse conditions. During the field experiments, amendment with BSFFF + NPK produced significantly (*p* < 0.001) bigger stem diameters than BSFFF at 4 and 6 weeks, BSG + NPK at 6 weeks, BSG, Evergrow, and NPK from the 6th to 10th week, and the control treatment from 4 to 10 weeks.

#### French Beans

The fertilizer treatments caused significant differences (greenhouse: *p* < 0.001, open field: *p* < 0.001) in the height, number of leaves, and stem diameter of French beans (Table 5). The plant height, number of leaves, and stem diameter increased to peak values at 7 weeks after planting. French beans grown using fertilizer treatments were significantly (*p* < 0.001) taller and had a higher number of leaves and bigger stem diameters compared to plants grown on unfertilized soil from the 4th week of experiments. During greenhouse experiments, soil amendment with BSFFF + NPK produced significantly (*p* < 0.001) taller French bean crops than NPK and control at 4 weeks, and other treatments from the 5th to 7th week of experiments, except where BSG + NPK was applied at 6 and 7 weeks and BSFFF at 7 weeks. In addition, treatment with BSFFF + NPK produced significantly (*p* < 0.001) taller plants from the 5th to 7th week of field experiments, except where BSG + NPK, NPK, and BSFFF were applied at 6 and 7 weeks.

**Table 5:** Effect of composted BSF frass, conventionally composted BSG, and commercial fertilizers on the growth of French beans.

**Parameter**	**Fertilizer treatment**	**Greenhouse**				**Open field**			
		**4 weeks**	**5 weeks**	**6 weeks**	**7 weeks**	**4 weeks**	**5 weeks**	**6 weeks**	**7 weeks**
Plant height (cm)	BSFFF	16.8 ± 2.58ab	25.1 ± 2.78bc	36.2 ± 1.54bc	38.2 ± 1.99a	12.8 ± 0.80a	19.4 ± 0.84b	24.3 ± 0.59a	26.9 ± 0.92ab
	BSG	16.8 ± 1.52ab	23.3 ± 2.85bc	30.7 ± 2.19c	32.5 ± 2.68b	9.1 ± 0.66b	14.9 ± 0.77c	19.2 ± 1.07b	21.5 ± 1.04c
	NPK	13.9 ± 2.46b	20.7 ± 2.61bc	29.7 ± 2.35c	31.8 ± 2.06b	14.5 ± 0.47a	19.4 ± 0.78b	24.0 ± 0.59a	26.6 ± 0.92ab
	Evergrow	14.6 ± 1.56ab	17.6 ± 1.75c	22.4 ± 1.58d	25.1 ± 1.31c	13.1 ± 0.64a	17.8 ± 0.70b	20.4 ± 0.79b	23.8 ± 0.88bc
	BSFFF + NPK	20.6 ± 0.55a	34.5 ± 0.71a	43.4 ± 1.66a	44.6 ± 1.44a	15.4 ± 0.41a	22.3 ± 1.26a	26.2 ± 1.34a	28.9 ± 1.53a
	BSG + NPK	19.4 ± 1.92ab	27.5 ± 2.94b	39.1 ± 2.85ab	40.6 ± 2.06a	14.8 ± 1.10a	19.0 ± 0.89b	24.1 ± 1.05a	27.2 ± 0.76ab
	Control	9.1 ± 0.92c	10.0 ± 1.07d	12.7 ± 1.22e	15.0 ± 1.14d	7.3 ± 0.58b	11.8 ± 0.68d	14.0 ± 0.99c	15.5 ± 0.67d
Number of leaves	BSFFF	5.3 ± 0.79a	8.6 ± 1.07ab	13.8 ± 0.66b	16.7 ± 0.78b	8.1 ± 0.80a	11.9 ± 1.12ab	13.9 ± 1.17ab	15.0 ± 1.08a
	BSG	4.9 ± 1.14a	8.1 ± 1.34ab	12.5 ± 0.53b	13.9 ± 1.13c	6.6 ± 0.85a	8.9 ± 0.81bc	10.9 ± 1.01bc	11.4 ± 0.88bc
	NPK	6.5 ± 1.16a	9.2 ± 1.6ab	13.8 ± 1.04b	15.9 ± 0.88b	8.3 ± 0.68a	11.2 ± 0.75ab	12.4 ± 0.78ab	13.8 ± 0.81ab
	Evergrow	4.0 ± 0.58a	6.0 ± 0.48bc	10.0 ± 1.09c	10.9 ± 1.0cd	7.2 ± 0.81a	9.3 ± 0.92bc	11.1 ± 1.00bc	11.8 ± 0.97bc
	BSFFF + NPK	6.5 ± 1.2a	11.6 ± 1.11a	17.9 ± 1.13a	19.9 ± 0.66a	8.9 ± 0.74a	13.5 ± 0.99a	15.6 ± 1.06a	16.9 ± 0.99a
	BSG + NPK	5.7 ± 0.73a	9.9 ± 1.14ab	15.2 ± 1.16b	17.9 ± 1.05a	8.4 ± 0.73a	11.9 ± 0.90ab	13.9 ± 0.98ab	15.6 ± 1.01a
	Control	3.0 ± 0.54a	3.7 ± 0.31c	4.6 ± 0.38d	5.0 ± 0.41d	5.9 ± 0.59a	7.3 ± 0.78c	8.1 ± 0.84c	8.7 ± 0.77c
Stem diameter (mm)	BSFFF	3.1 ± 0.06ab	4.9 ± 0.46abc	5.5 ± 0.39abc	6.8 ± 0.24a	3.2 ± 0.22ab	4.4 ± 0.23ab	5.4 ± 0.32ab	6.3 ± 0.36ab
	BSG	2.9 ± 0.16ab	3.9 ± 0.32bcd	4.4 ± 0.41bcd	5.4 ± 0.35b	3.0 ± 0.20ab	3.8 ± 0.27b	4.6 ± 0.37b	5.2 ± 0.40bc
	NPK	3.2 ± 0.51ab	4.8 ± 0.24abc	5.2 ± 0.24abc	5.6 ± 0.12b	3.3 ± 0.23ab	4.3 ± 0.21ab	4.9 ± 0.19ab	5.5 ± 0.18bc
	Evergrow	2.5 ± 0.25ab	3.3 ± 0.58cd	4.0 ± 0.73cd	4.3 ± 0.7b	2.7 ± 0.23bc	3.5 ± 0.39b	4.2 ± 0.48b	4.6 ± 0.47c
	BSFFF + NPK	3.6 ± 0.33a	5.7 ± 0.23a	6.6 ± 0.20a	7.6 ± 0.19a	3.8 ± 0.25a	5.0 ± 0.29a	6.1 ± 0.23a	6.8 ± 0.17a
	BSG + NPK	2.8 ± 0.36ab	5.2 ± 0.55ab	5.9 ± 0.46ab	6.9 ± 0.2a	3.0 ± 0.24ab	4.5 ± 0.34ab	5.4 ± 0.37ab	6.2 ± 0.31ab
	Control	2.0 ± 0.19b	2.5 ± 0.39d	2.9 ± 0.48d	3.1 ± 0.46c	2.1 ± 0.15c	2.7 ± 0.28c	3.1 ± 0.30c	3.3 ± 0.30d

*BSFFF, black soldier fly frass organic fertilizer applied at sole rate equivalent to 371 kg N ha^−1^; BSG, conventional compost from brewer's spent grain applied at sole rate equivalent to 371 kg N ha^−1^; NPK, commercial mineral fertilizer applied at sole rate equivalent to 371 kg N ha^−1^; Evergrow, commercial organic fertilizer applied at sole rate equivalent to 371 kg N ha^−1^; BSFFF + NPK, combined application of BSFFF and NPK so that each supplies 185.5 kg N ha^−1^; BSG + NPK, combined BSG compost and NPK so that each supplies 185.5 kg N ha^−1^, control, unfertilized soil. Within the same column and per parameter, means (±SE) followed by the same letters are not significantly different for α = 0.05*.

During greenhouse experiments, soil amendment with BSFFF + NPK produced significantly (*p* < 0.001) higher number of leaves than Evergrow, and control treatments at 4 weeks and other treatments from the 6th to 7th week, except where BSG + NPK was applied at 7 weeks. Likewise, the number of leaves produced by French bean grown using BSFFF + NPK was significantly (*p* < 0.001) higher than those achieved using BSG, Evergrow, and control treatments from the 5th to 7th week during open-field experiments.

It was noted that application of BSFFF + NPK significantly (*p* < 0.001) increased French bean stem diameter than BSG, Evergrow, and control treatments from the 5th to 7th week of both experiments, and NPK at 7 weeks during both greenhouse and field experiments. At 7 weeks, treatment with BSFFF frass produced significantly (*p* < 0.001) bigger French bean stem diameter than BSG and NPK during greenhouse experiments, and Evergrow during both experiments.

### Vegetable Yields

#### Tomatoes

**Figure 1:**
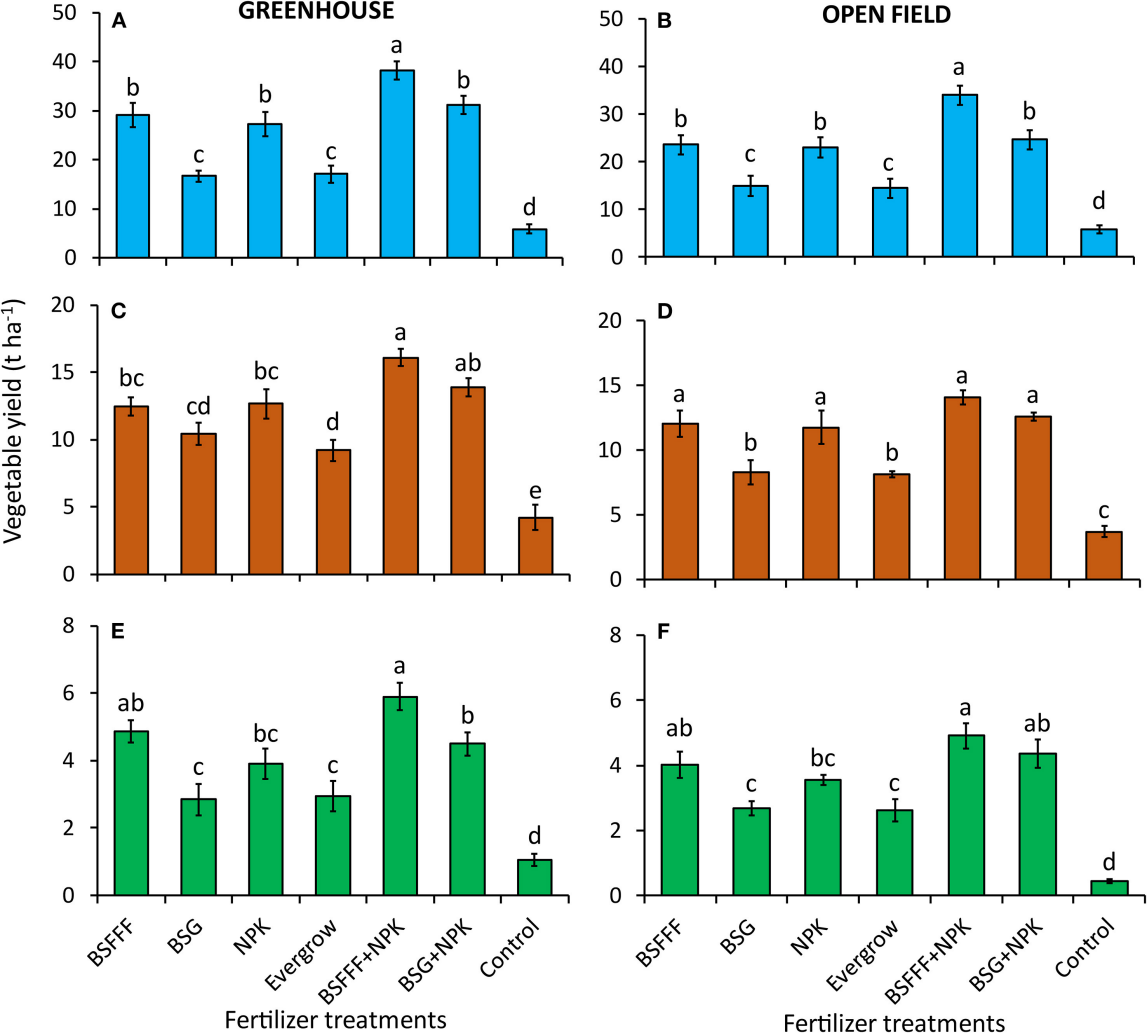
Effect of composted BSF frass fertilizer, conventionally composted BSG, and commercial organic and inorganic fertilizer on the yield of tomatoes **(A,B)**, kales **(C,D)**, and French beans **(E,F)** under greenhouse **(A,C,E)** and open-field **(B,D,F)** conditions. BSFFF, black soldier fly frass organic fertilizer applied at sole rate equivalent to 371 kg N ha^−1^; BSG, conventional compost from brewer's spent grain applied at sole rate equivalent to 371 kg N ha^−1^; NPK, commercial mineral fertilizer applied at sole rate equivalent to 371 kg N ha^−1^; Evergrow, commercial organic fertilizer applied at sole rate equivalent to 371 kg N ha^−1^; BSFFF + NPK, combined application of BSFFF and NPK so that each supplies 185.5 kg N ha^−1^; BSG + NPK, combined BSG compost and NPK so that each supplies 185.5 kg N ha^−1^, control, unfertilized soil. Per panel, means (±SE) followed by the same letters are not significantly different for α = 0.05.

There were significant differences in the yield of tomato grown using different fertilizer treatments (greenhouse: *p* < 0.001, open field: *p* < 0.001). The tomato yields achieved using fertilizer treatments were significantly (*p* < 0.001) higher than those obtained using unfertilized soil by 2.9–6.5 and 2.6–6.1 times under greenhouse and open-field conditions, respectively (Figures 1A,B). Amendment with BSFFF + NPK produced the highest tomato yields compared to the other fertilizer treatments, which were 22–124% and 38–135% higher under greenhouse (*p* < 0.001) and open-field (*p* < 0.001) conditions, respectively. The tomato yields achieved using sole BSFFF were significantly (*p* < 0.001) higher than those where BSG (58–75%) and Evergrow (63–71%) were applied.

#### Kales

The yield of kale plants grown using different fertilizer treatments varied significantly during experiments (greenhouse: *p* < 0.001, open field: *p* < 0.001). Kales grown using fertilizer treatments yielded significantly higher than those grown on unfertilized soil (Figures 1C,D). Amendment with BSFFF + NPK fertilizer produced significantly (*p* < 0.001) higher than those of BSG and Evergrow under both greenhouse and open-field conditions, and BSFFF and NPK during greenhouse experiments. During the greenhouse experiments, kale yields from pots treated with BSFFF, NPK, and BSG + NPK were significantly (*p* < 0.001) higher than that of Evergrow.

#### French Beans

The pod yields of French beans were significantly influenced by different fertilizer amendments under both greenhouse (*p* < 0.001) and open-field (*p* < 0.001) conditions. All fertilizer treatments produced a significantly (*p* < 0.001) higher French bean yield than the control. Treatment BSFFF + NPK produced significantly (*p* < 0.001) higher French bean yields than those of other treatments, except BSFFF under both production environments and where BSG + NPK was applied under open-field conditions (Figures 1E,F). The French bean yields achieved using BSFFF and BSG + NPK under greenhouse conditions were significantly (*p* < 0.001) higher than those of BSG and Evergrow. Furthermore, soil treated with BSFFF, NPK, and BSG + NPK produced significantly (*p* < 0.001) higher French bean yields under open-field conditions compared to amendment with BSG and Evergrow.

The yields of French beans achieved using BSFFF were 70, 24, and 65% higher than those of BSG, NPK, and Evergrow, respectively, during greenhouse experiments. In addition, soil treated with BSFFF produced 50, 14, and 52% higher French bean yields than BSG, NPK, and Evergrow, respectively, during open-field experiments. Amendment with BSFFF + NPK produced 31% higher yield than BSG + NPK under greenhouse conditions. Likewise, French bean yield obtained using BSFFF + NPK was 50 and 38% higher than those achieved using NPK during greenhouse and open-field experiments, respectively.

### Nitrogen Uptake

The fertilizer amendments caused significant variation in the N uptake of tomatoes (*p* < 0.001), kales (*p* < 0.001), and French beans (*p* < 0.001) (Table 6). Crops grown using fertilizer treatments accumulated significantly (*p* < 0.001) higher N than the control treatment. It was noted that tomatoes and kales grown using BSFFF + NPK accumulated significantly (*p* < 0.001) higher N than other treatments. The N accumulated by tomatoes and kales grown using BSFFF, NPK, and BSG + NPK was significantly (*p* < 0.001) higher than those of tomatoes grown in soil amended with BSG or Evergrow.

**Table 6:** Nitrogen uptake and agronomic nitrogen use efficiency of tomatoes, kales, and French beans grown using composted BSF frass, conventionally composted BSG, and commercial fertilizers.

**Parameter**	**Fertilizer treatment**	**Tomatoes**	**Kales**	**French beans**
Nitrogen uptake (kg N ha^−1^)	BSFFF	45.6 ± 7.14b	254.2 ± 30.89b	97.5 ± 11.82b
	BSG	17.6 ± 4.12cd	164.7 ± 7.75c	57.4 ± 2.39c
	NPK	41.9 ± 4.68b	255.8 ± 12.13b	107.7 ± 7.63b
	Evergrow	23.7 ± 5.9c	160.9 ± 5.23c	55.9 ± 3.01c
	BSFFF + NPK	65.9 ± 3.33a	376.3 ± 13.61a	160.4 ± 15.48a
	BSG + NPK	47.2 ± 7.14b	295.3 ± 32.95b	137.9 ± 10.04a
	Control	3.8 ± 0.65d	43.1 ± 7.96d	7.9 ± 1.29d
Agronomic nitrogen use efficiency (kg kg^−1^ N)	BSFFF	118.4 ± 10.26b	55.7 ± 5.38a	23.8 ± 3.17ab
	BSG	60.5 ± 11.05c	30.6 ± 3.67b	15 ± 1.81c
	NPK	114.6 ± 10.64b	53.6 ± 6.22a	20.7 ± 1.39bc
	Evergrow	57.4 ± 10.14c	29.6 ± 4.03b	14.6 ± 1.93c
	BSFFF + NPK	187.6 ± 10.16a	69.4 ± 2.9a	29.7 ± 2.23a
	BSG + NPK	125.4 ± 10.89b	59.4 ± 4.74a	26.2 ± 2.56ab

*BSFFF, black soldier fly frass organic fertilizer applied at sole rate equivalent to 371 kg N ha^−1^; BSG, conventional compost from brewer's spent grain applied at sole rate equivalent to 371 kg N ha^−1^; NPK, commercial mineral fertilizer applied at sole rate equivalent to 371 kg N ha^−1^; Evergrow, commercial organic fertilizer applied at sole rate equivalent to 371 kg N ha^−1^; BSFFF + NPK, combined application of BSFFF and NPK so that each supplies 185.5 kg N ha^−1^; BSG + NPK, combined BSG compost and NPK so that each supplies 185.5 kg N ha^−1^, control, unfertilized soil. Within the same column and per parameter, means (±SE) followed by the same letters are not significantly different for α = 0.05*.

Use of sole BSFFF increased N uptake in tomatoes by 91 and 161% compared to where Evergrow or BSG were applied, respectively (Table 6). In addition, tomatoes grown using BSFFF + NPK accumulated 40, 44, and 58% higher N than where BSG + NPK, BSFFF, and NPK were applied, respectively. The N accumulated in kales grown using BSFFF was 54 and 59% higher than those accumulated by BSG and Evergrow, respectively. Amendment with BSFFF + NPK increased the N uptake of kales by 47 and 27% compared to NPK and BSG + NPK, respectively.

The French beans grown using BSFFF + NPK or BSG + NPK accumulated significantly (*p* < 0.001) higher N than other treatments (Table 6). Similarly, soil amendment with BSFFF or NPK caused significantly higher N accumulation in French beans than where BSG and Evergrow were applied. However, the N accumulated in French beans grown using BSFFF + NPK was 49% higher than that of the crops grown in soil amended with NPK. Furthermore, soil treated with BSFFF produced French beans with 70 and 75% higher N uptake than where BSG and Evergrow were applied, respectively.

### Agronomic N Use Efficiency

There were significant (*p* < 0.001) differences in the AE_N_ of tomatoes, kales, and French beans grown using various fertilizer treatments (Table 6). Tomatoes grown in soil amended with either BSFFF + NPK or BSG + NPK achieved significantly (*p* < 0.001) higher AE_N_ values than other treatments. In addition, the AE_N_ of tomatoes grown using BSFFF and NPK were significantly (*p* < 0.001) higher than those of BSG and Evergrow treatments. Similarly, amendment with BSFFF increased the AE_N_ of tomatoes by 96 and 106% compared to where BSG and Evergrow were applied, respectively.

The AE_N_ of kales grown using Evergrow and BSG compost was significantly (*p* < 0.001) lower than those of other treatments (Table 6). Amendment with combined BSFFF + NPK produced tomatoes with the highest AE_N_, which was 29% higher than that achieved using NPK. Furthermore, the AE_N_ kales grown in soil treated with sole BSF frass were 82 and 89% higher than those of kales grown using BSG and Evergrow fertilizers, respectively. It was noted that French beans grown in soil treated with BSFFF + NPK had the highest AE_N_, which was two times higher (*p* < 0.001) than those of French beans grown using BSG and Evergrow. Likewise, the application of BSFFF and BSG + NPK produced French beans with significantly (*p* < 0.001) higher AE_N_ values than where BSG and Evergrow were applied.

### Nutritional Quality of Vegetables Grown Using Different Fertilizers

#### Tomatoes

The different fertilizer treatments caused significant differences in the ash (*p* < 0.001), crude fat (*p* < 0.001), crude protein (*p* < 0.001), crude fiber (*p* < 0.001), and carbohydrate (*p* < 0.001) concentrations of tomatoes (Table 7). Amendment with BSFFF + NPK produced tomatoes with the highest crude protein, significantly higher (*p* < 0.001) than that of tomatoes grown using BSG. In addition, amendment with BSFFF achieved significantly higher crude protein in tomatoes than where BSG was applied. Furthermore, the highest ash concentration was recorded in tomatoes grown using BSFFF + NPK, and this was significantly (*p* < 0.001) higher than those of BSG and Evergrow.

**Table 7:** Influence of composted BSF frass, conventionally composted BSG, and commercial fertilizers on the nutritional quality of tomatoes, kales, and French beans.

**Crop**	**Fertilizer treatment**	**Ash**	**Crude fat**	**Crude protein**	**Crude fiber**	**Carbohydrates**
		**(%)**				
Tomatoes	BSFFF	0.41 ± 0.3ab	0.23 ± 0.01cd	1.19 ± 0.08a	1.26 ± 0.06c	4.7 ± 0.70bc
	BSG	0.23 ± 0.04cd	0.19 ± 0.02cd	0.71 ± 0.10b	1.61 ± 0.05a	5.3 ± 0.56b
	NPK	0.45 ± 0.04a	0.28 ± 0.03c	1.13 ± 0.04a	1.36 ± 0.07bc	2.9 ± 0.36c
	Evergrow	0.32 ± 0.02bc	0.12 ± 0.02d	0.98 ± 0.13a	1.63 ± 0.05a	4.6 ± 0.63bc
	BSFFF + NPK	0.48 ± 0.04a	0.37 ± 0.04b	1.22 ± 0.08a	1.40 ± 0.06bc	3.9 ± 0.38bc
	BSG + NPK	0.53 ± 0.05a	0.49 ± 0.05a	1.18 ± 0.08a	1.52 ± 0.07ab	3.0 ± 0.56c
	Control	0.17 ± 0.01d	0.15 ± 0.02d	0.42 ± 0.06c	1.32 ± 0.04c	6.9 ± 0.48a
Kales	BSFFF	10.5 ± 1.10a	3.9 ± 0.11a	13.2 ± 1.08a	12.1 ± 0.47a	47.4 ± 2.78b
	BSG	5.7 ± 0.38b	3.9 ± 0.15a	12.8 ± 1.14a	11.2 ± 0.37a	54.3 ± 1.89b
	NPK	10.1 ± 1.01a	3.9 ± 0.19a	14.0 ± 1.23a	12.3 ± 0.34a	47.0 ± 3.23b
	Evergrow	6.2 ± 0.31b	3.5 ± 0.23a	12.4 ± 0.20a	11.9 ± 0.48a	53.1 ± 1.00b
	BBSFFF + NPK	9.8 ± 1.01a	3.6 ± 0.13a	16.7 ± 0.54a	11.2 ± 0.49a	45.4 ± 2.22b
	BSG + NPK	9.7 ± 1.07a	3.7 ± 0.18a	14.6 ± 1.27a	12.1 ± 0.42a	46.2 ± 3.00b
	Control	4.1 ± 0.31b	2.4 ± 0.16b	7.4 ± 1.20b	10.9 ± 0.41a	65.2 ± 1.61a
French beans	BSFFF	7.2 ± 0.24ab	1.5 ± 0.14bc	15.1 ± 0.85b	13.3 ± 1.10ab	49.8 ± 1.64bc
	BSG	5.0 ± 0.20c	1.1 ± 0.26cd	13.5 ± 0.69bc	14.6 ± 0.86a	54.6 ± 0.68b
	NPK	7.7 ± 0.76ab	1.7 ± 0.06bc	18.9 ± 0.74a	13.3 ± 0.91ab	45.9 ± 2.27cd
	Evergrow	5.6 ± 0.18bc	1.5 ± 0.18bc	13.7 ± 0.99bc	13.4 ± 1.08ab	55.8 ± 2.06b
	BSFFF + NPK	9.1 ± 1.08a	2.4 ± 0.20a	20.4 ± 1.03a	14.3 ± 0.63a	40.8 ± 0.90d
	BSG + NPK	8.6 ± 0.51a	2.0 ± 0.19ab	19.9 ± 0.81a	15.9 ± 1.13a	40.9 ± 2.65d
	Control	4.6 ± 0.60c	0.8 ± 0.10d	11.2 ± 0.63c	9.7 ± 0.61b	62.5 ± 1.16a

*BSFFF, black soldier fly frass organic fertilizer applied at sole rate equivalent to 371 kg N ha^−1^; BSG, conventional compost from brewer's spent grain applied at sole rate equivalent to 371 kg N ha^−1^; NPK, commercial mineral fertilizer applied at sole rate equivalent to 371 kg N ha^−1^; Evergrow, commercial organic fertilizer applied at sole rate equivalent to 371 kg N ha^−1^; BSFFF + NPK, combined application of BSFFF and NPK so that each supplies 185.5 kg N ha^−1^; BSG + NPK, combined BSG compost and NPK so that each supplies 185.5 kg N ha^−1^, control, unfertilized soil. Within the same column and per parameter, means (±SE) followed by the same letters are not significantly different for α = 0.05*.

Tomatoes grown using BSG + NPK had significantly (*p* < 0.001) higher crude fat concentration than other treatments (Table 7). Furthermore, amendment with BSFFF or NPK-produced tomatoes with the significantly (*p* < 0.001) higher crude fat concentration than other treatments, except combined BSFFF + NPK. The crude fat concentration of tomatoes harvested from plots amended with NPK was significantly (*p* < 0.001) higher than those of tomatoes grown using BSFFF, BSG, and Evergrow. Tomatoes grown using Evergrow, BSG, and BSG + NPK achieved significantly (*p* < 0.001) higher crude fiber concentrations than other treatments. The carbohydrate concentration of tomatoes grown using unfertilized soil (control) was significantly (*p* < 0.001) higher than those of other treatments.

#### Kales

The ash (*p* < 0.001), crude fat (*p* < 0.001), crude protein (*p* < 0.001), and carbohydrate (*p* < 0.001) concentrations of kales also varied significantly due to different fertilizer amendments (Table 7). The ash concentration recorded in kales grown using BSFFF, and this was significantly (*p* < 0.001) higher than those of kales grown using BSG, Evergrow, and the control treatment. The crude fat concentrations of kales grown in fertilizer-treated soils were significantly (*p* < 0.001) higher than that of kale grown using unfertilized soil.

Amendment with fertilizers produced kales with significantly (*p* < 0.001) higher crude fat and crude protein concentrations than the control treatment (Table 7). Amendment with BSFFF + NPK doubled the crude protein concentration of kales compared to the control. However, there were no significant differences in the crude protein concentrations of kales grown using different fertilizer treatments. Moreover, the crude fiber concentration of kales did not vary significantly during experiments. The control treatment produced kales with significantly (*p* < 0.001) higher carbohydrate concentration than other treatments.

#### French Beans

The ash (*p* < 0.001), crude fat (*p* < 0.001), crude protein (*p* < 0.001), crude fiber, and carbohydrate (*p* < 0.001) concentrations of French beans grown using different fertilizer amendments varied significantly (Table 7). The highest ash concentration of French beans grown using BSFFF + NPK was significantly (*p* < 0.001) higher than those of BSG, Evergrow, and control treatments. In addition, soil amended with BSFFF produced French beans with significantly (*p* < 0.001) higher ash concentrations than the control and BSG. Furthermore, the crude fat concentration of French beans grown using BSFFF + NPK was significantly (*p* < 0.001) higher than those of other treatments, except where BSG + NPK was applied.

The crude protein of French beans produced using BSFFF + NPK, BSG + NPK, NPK, and BSFFF were significantly (*p* < 0.001) higher than those achieved using the control treatment by 82, 78, 69, and 35%, respectively (Table 7). Moreover, amendment with BSFFF + NPK, BSG + NPK, and NPK produced French beans with significantly (*p* < 0.001) higher crude protein concentrations than other treatments. The crude fiber concentrations of French beans grown in soil treated with BSFFF + NPK, BSG + NPK, and BSG were significantly (*p* < 0.001) higher than that achieved using the control treatment.

The carbohydrate concentration of French harvested from the unfertilized soil was significantly (*p* < 0.001) higher than those of other treatments (Table 7). Furthermore, soil treatment with BSG and Evergrow fertilizers produced French beans with significantly (*p* < 0.001) higher carbohydrate concentration than where NPK, BSFFF + NPK, and BSG + NPK were applied. The carbohydrate concentration of French beans treated with BSFFF or BSG was significantly (*p* < 0.001) higher than those of French beans grown using BSFFF + NPK and BSG + NPK.

### Multivariate Analysis of Vegetable Growth, Yield, and Nutritional Quality

**Figure 2:**
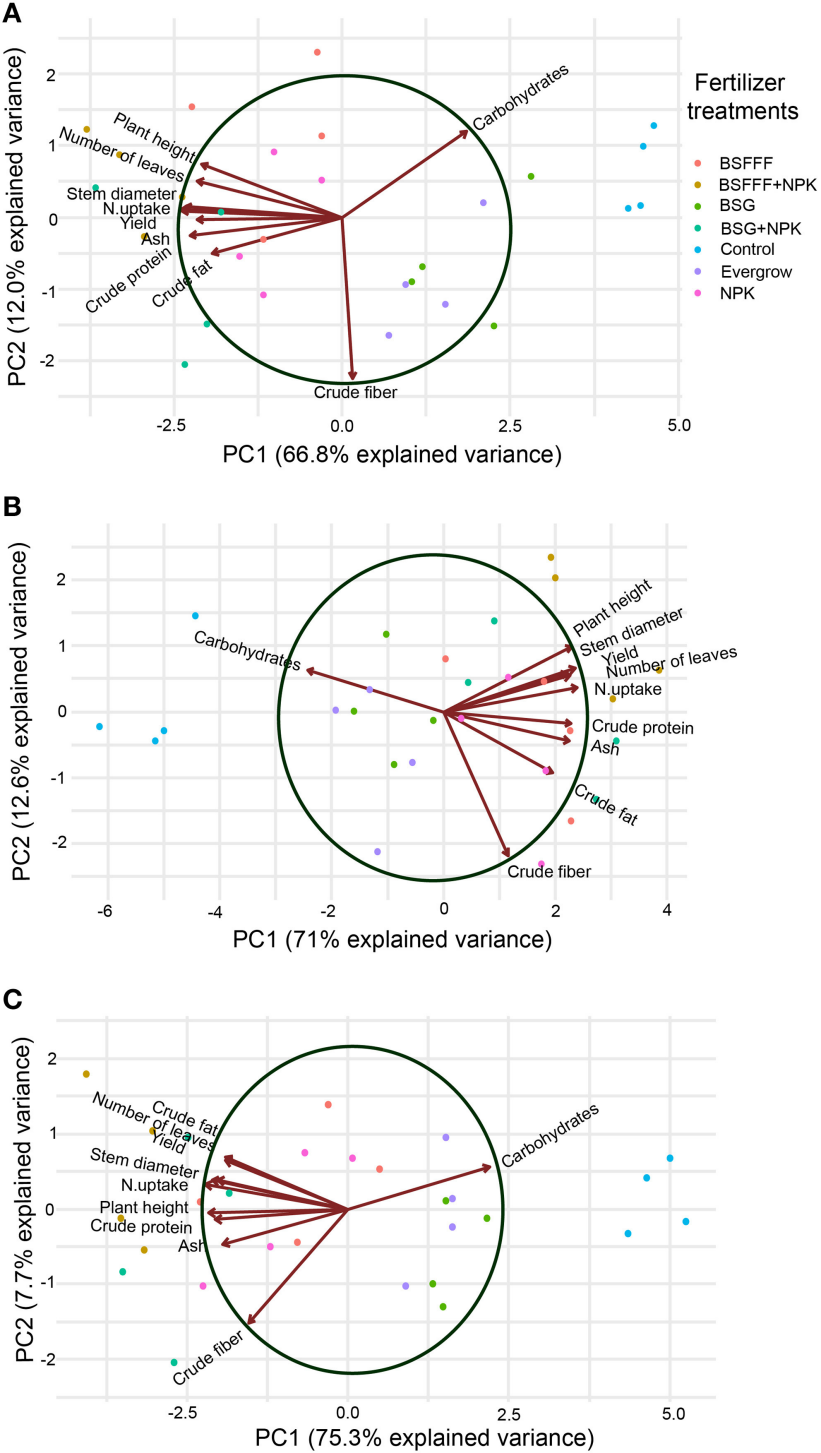
Biplot graphs based on the PC analysis of parameters that measure **(A)** tomato, **(B)** kale and **(C)** French bean growth, yield, and nutritional quality as a function of the different fertilizer treatments. BSFFF, black soldier fly frass organic fertilizer applied at sole rate equivalent to 371 kg N ha^−1^; BSG, conventional compost from brewer's spent grain applied at sole rate equivalent to 371 kg N ha^−1^; NPK, commercial mineral fertilizer applied at sole rate equivalent to 371 kg N ha^−1^; Evergrow, commercial organic fertilizer applied at sole rate equivalent to 371 kg N ha^−1^; BSFFF + NPK, combined application of BSFFF and NPK so that each supplies 185.5 kg N ha^−1^; BSG + NPK, combined BSG compost and NPK so that each supplies 185.5 kg N ha^−1^, control, unfertilized soil.

The PCA revealed that the growth, yield, and nutritional quality of the three vegetables were significantly affected by fertilizer amendments (Figure 2). For tomatoes, the first two components of PCA explained 78.8% of the total data variance with PC 1 and PC 2 accounting for 66.8 and 12.0%, respectively (Figure 2A). It was noted that tomato height, number of leaves, stem diameter, and N uptake were positively correlated, whereas tomato yield, crude protein, ash concentration, and crude fat were negatively correlated. However, carbohydrates and crude fiber were negatively correlated with the rest of the parameters.

The first two components of the PCA explained 82.6% of the total variance in kale growth, yield, and nutritional quality, whereby PC 1 accounted for 71% and PC 2 accounted for 12.6% of the total variance (Figure 2B). The kale height, stem diameter, number of leaves, N uptake, and yield were positively correlated. The crude protein, ash, crude fat, and crude fiber were positively correlated in PC 1. For French beans, first two components of PCA explained 83% of the total variance, PC 1 and PC 2 accounted for 75.3 and 7.7% of the total variance, respectively (Figure 2C). The French bean yield, N uptake, stem diameter, number of leaves, and crude fat were positively correlated. However, crude protein, crude fiber, and ash were negatively correlated. For both kales (Figure 2B) and French beans (Figure 2C), it was noted that carbohydrate concentration was negatively correlated with the rest of the parameters.

## Discussion

Most research efforts on vegetable production in SSA have focused on the reduction of pest and disease pressure, weeds, and the mitigation of climatic hazards such as excessive solar irradiation, rain, and wind, using physical protection (Nordey et al., 2017). However, little has been done to develop cost-effective and environmentally friendly and sustainable fertilizer products for improving vegetable growth and yield. The higher growth, yield, and nutritional quality of three vegetables grown using fertilizers compared to unfertilized soil, indicate high levels of nutrient depletion in the experimental soils (Table 1) and in most regions of SSA (Gachimbi et al., 2005; Nkonya et al., 2008; Tully et al., 2015; Wortmann et al., 2019). The current study provides evidence on the high efficiency of composted BSF frass as fertilizer for improving the growth, yield, and nutritional quality of vegetable crops.

The composted BSF frass and its combination with NPK fertilizer performed better than other treatments on increasing the height, leaf growth, and stem diameter of tomatoes, kales, and French beans (Figure 1). The higher growth rate and yield associated with vegetables grown using BSFFF could also be attributed to its high maturity and stability as indicated by parameters such as the lower C/N ratio (11) and high germination index (86%) (Table 2). Findings from this study are in line with Beesigamukama et al. (2020a, 2021) who reported the absence of phytotoxicity in composted BSF frass as organic fertilizer. Therefore, the increased vegetable growth, N uptake in vegetables grown using BSFFF compared to the conventional compost, and commercial organic and mineral fertilizers could be attributed to better supply and availability of nutrients from the newly introduced frass fertilizer (Beesigamukama et al., 2020b). Furthermore, the high release of nutrients resulting from the high mineralization rate of BSFFF (Adin Yéton et al., 2019) might have partly contributed to better synchrony of nutrient supply for vegetables and high yields.

The higher vegetable growth, yield, and nutritional quality achieved using a combination of BSF frass and NPK could be attributed to the synergistic and complementary effects of organic and inorganic fertilizers on crop growth and yield have been reported previously (Boateng et al., 2009; Nsoanya and Nweke, 2015; Islam et al., 2017; Musyoka et al., 2017). The current study agrees with earlier findings of Islam et al. (2017) who reported that the combined use of organic and inorganic fertilizer increases the growth and yield of tomatoes.

The low growth rate and yields associated with the conventional compost (BSG) could be due to moderate phytotoxicity as indicated by the low seed germination index (Table 2). The lower growth rate observed in soils treated with conventional compost (BSG) and commercial organic fertilizer (Evergrow) could have been caused by the higher C/N ratios associated with these organic fertilizers (Table 2). Our findings agree with those of Beesigamukama et al. (2020b) and Palomba (2016) who reported higher crop yields and faster nutrient release while using organic fertilizers with low C/N ratios. The higher vegetable yields achieved under greenhouse than open-field conditions could be largely attributed to the presence of ideal conditions for plant growth (water, temperature, minimal loss of nutrients, and absence of pests and diseases) provided by the greenhouse environment.

The higher crude protein, fat, fiber, and ash concentrations achieved using composted BSF frass as fertilizer indicate that in addition to enhancing vegetable yields, the BSFFF is effective in increasing the nutritional quality of vegetable crops. The higher crude protein, fat, fiber, and ash concentrations achieved using BSFFF (Table 7, Figure 2) are comparable to values reported in previous studies (van der Lans et al., 2012; Chakwizira et al., 2015; Dunsin et al., 2016; Mukta et al., 2016; Meena et al., 2018; Yoder and Davis, 2020) and could be attributed to the high uptake of N (Table 6) and other nutrients by crops grown using this organic fertilizer (Beesigamukama et al., 2020b; Menino et al., 2021). Information on the nutritional quality of vegetable crops grown using BSFFF is still scarce. Therefore, the current study acts as a benchmark for future research efforts.

## Conclusion

This study provides clear evidence that soil amendment with composted BSF frass as an organic fertilizer has the potential to create favorable soil conditions for vegetable cultivation leading to improved performance. Sole application of composted BSF as organic fertilizer or its integration with NPK fertilizer boosts the growth, yield, and nutritional quality similarly or better than the commercial organic and mineral fertilizers. Therefore, the application rate of 2.5 t ha^−1^ BSFFF or combined application of 1.24 t ha^−1^ BSF-composted organic fertilizer with 322 kg ha^−1^ of NPK is recommended for improving the production of tomatoes, kales, and French beans. Adoption of high-quality fertilizer products, such as the BSF-composted organic fertilizer, would reduce the use of expensive mineral fertilizers and consequently lessen the associated burden of environmental pollution. More field experiments should be carried out in different agroecological zones for a wider application of the current research findings.

## Data Availability Statement

The original contributions presented in the study are included in the article/supplementary material, further inquiries can be directed to the corresponding author/s.

## Author Contributions

AA, NK, and CT: conceptualization. AA, NK, DB, and CT: data curation, formal analysis, and methodology. JL, MD, and CT: funding acquisition. AA, NK, DB, KN, and CT: investigation. CT: project administration. NK, KN, and CT: resources. AA, DB, and CT: software, validation, and visualization. NK and CT: supervision. AA, GC, DB, and CT: writing and original draft preparation. AA, NK, JL, MD, DB, KN, GC, SS, and CT: writing—review and editing. All authors contributed to the article and approved the submitted version.

## Conflict of Interest

The authors declare that the research was conducted in the absence of any commercial or financial relationships that could be construed as a potential conflict of interest.
